# Towards an EU measure of child deprivation

**DOI:** 10.1007/s12187-017-9491-6

**Published:** 2017-10-10

**Authors:** Anne-Catherine Guio, David Gordon, Eric Marlier, Hector Najera, Marco Pomati

**Affiliations:** 1 Luxembourg Institute of Socio-Economic Research (LISER), Esch-sur-Alzette, Luxembourg; 2 University of Bristol, Bristol, UK; 3 Cardiff University, Cardiff, UK

**Keywords:** Child deprivation, Poverty, European Union, Monitoring, Europe 2020

## Abstract

This paper proposes a new measure of child material and social deprivation (MSD) in the European Union (EU) which includes age appropriate child-specific information available from the thematic deprivation modules included in the 2009 and 2014 waves of the “EU Statistics on Income and Living Conditions” (EU-SILC). It summarises the main results of the in-depth analysis of these two datasets, identifies an optimal set of robust children MSD items and recommends a child-specific MSD indicator for use by EU countries and the European Commission in their regular social monitoring. In doing this, the paper replicates and expands on the methodological framework outlined in Guio et al. (2012, 2016), particularly by including additional advanced reliability tests.

## Introduction

On the 25th of September 2015, the 194 United Nations Member States adopted 17 Sustainable Development Goals designed to guide social, economic and environmental policy in all countries (including EU countries) between 2015 and 2030. Goal 1 is about ending poverty in all its forms everywhere and Target 1.2 consists of, by 2030, reducing “*at least by half the proportion of men, women and children of all ages living in poverty in all its dimensions according to national definitions*”. It is the first time that the governments of the world have agreed on a multidimensional poverty target which explicitly includes children. The previous Millennium Development Goals did not specifically mention child poverty. The work of UNICEF has played a major role in moving the issue of child poverty and social exclusion and also that of child well-being up the global social agenda (see, for instance, Bradshaw et al. 2006; UNICEF 2012 and 2013 and also the UNICEF’s first *Global Study of Child Poverty and Disparities* 1).

The work of the European Union (EU) was also of importance in this dynamic. The fight against child poverty and social exclusion and the importance of investing in children’s well-being has been high on the EU policy agenda for more than a decade. A first significant step was the independent report on *Taking forward the EU Social Inclusion Process*, commissioned by the EU Luxembourg Presidency in the first half of 2005, subsequently updated and published as Marlier et al. (2007). This report stressed the need for “children mainstreaming” and suggested a specific approach to child well-being at EU level. It also argued that simple age group breakdowns of EU social indicators were insufficient to adequately capture the multi-dimensional nature of poverty and social exclusion of children – child-specific measures are needed. Following this recommendation, the EU Social Protection Committee (SPC) decided to reserve a slot for (at least) one indicator on “child well-being” in the EU portfolio of social protection and social inclusion indicators^2^ and to set up an *EU Task-Force on Child Poverty and Child Well-Being*. The report of this Task-Force and its 15 recommendations were endorsed by the European Commission and all EU countries in 2008 (Social Protection Committee 2008).^3^ Another step forward was taken in February 2013, when the European Commission published a Recommendation on “Investing in children, breaking the cycle of disadvantage”, which was also endorsed by all EU Member States a few months later (European Commission 2013; see also Frazer and Marlier 2014, 2017). The Commission’s Recommendation builds on research commissioned by three EU Presidencies that took place between 2010 and 2012,^4^ as well as research (commissioned) by the SPC and/or the European Commission (Belgian Presidency of the European Union 2010; Frazer et al. 2010; Tárki and Applica 2010; Tárki 2011; Frazer and Marlier 2012; SPC 2012).

Both the needs and living standards of children can be different from those of adults, even within the same households (Gordon and Nandy 2012; Main and Besemer 2013; Dermott and Pomati 2016; Main and Bradshaw 2016). Thus, although many of the household level material and social deprivation (MSD) items available from the core questionnaire of the *EU Statistics on Income and Living Conditions (EU-SILC)* are relevant to the situation of children, the accurate measurement of the actual living conditions of children requires the collection of information specific to the children’s situation and needs. The 2009 EU-SILC ad hoc module on deprivation included child-specific MSD items, which made it possible to develop specific child MSD indicators (see Gábos et al. 2011; Guio et al. 2012; de Neubourg et al. 2012; Watson et al. 2012; Whelan 2012). The 13 child-specific items which passed the robustness analysis performed by Guio et al. (2012) were collected for a second time in the 2014 ad hoc EU-SILC module on deprivation in the whole EU and in a number of non-EU countries that also carry out the EU-SILC. This paper provides the main results of the in-depth analysis of the 2014 EU-SILC data. It replicates and expands the original analysis which Guio et al. (2012) performed on the 2009 data. A key purpose of the paper is to identify an optimal set of children MSD items in order to recommend a child MSD indicator for use by EU Member States and the European Commission in their regular social monitoring.

The paper is organised as follows: Section 2 describes the methodological framework, Section 3 presents the child MSD items which are tested against this framework, Sections 4 to 7 summarise the results of the various robustness tests (suitability, validity, reliability and additivity) and, finally, Section 8 proposes an aggregate indicator and Section 9 concludes.

## Methodological Framework

The conceptual approach followed in this paper is inspired by Peter Townsend’s relative deprivation research during the 1960s and succinctly described in 1979 as follows:

“*Poverty can be defined objectively and applied consistently only in terms of the concept of relative deprivation. […] Individuals, families and groups in the population can be said to be in poverty when they lack the resources to obtain the type of diet, participate in the activities and have the living conditions and amenities which are customary, or at least widely encouraged or approved, in the societies to which they belong. Their resources are so seriously below those commanded by the average individual or family that they are, in effect, excluded from ordinary living patterns, customs or activities*.” (Townsend 1979, p. 31)

Our analytical framework draws extensively on the 1999 *Poverty and Social Exclusion (PSE) Survey* deprivation indicator construction methodology (Gordon et al. 2000; Pantazis et al. 2006a, b). This methodology has been used to develop robust and comparable measures of deprivation for various poverty surveys (see for example Hillyard et al. 2003; Gordon 2010; Fahmy et al. 2011). An important aspect of this methodology is that it facilitates the identification and selection of an optimal set of MSD items from the initial list of available items. This framework was also used at EU level (see Guio et al. 2012, 2016 and 2017).

So, to identify the final optimal list of MSD items we considered four aspects in turn:

1. The *suitability* of each MSD item, in order to check that respondents in the different Member States (as well as the different population sub-groups within each Member State) consider them necessary to have an “acceptable” standard of living in the country where they live. Here, “suitability” is understood as a measure of “face validity” amongst the EU population.
2. The *validity* of individual items, to ensure that each item exhibits statistically significant relative risk ratios with independent variables known to be correlated with MSD.
3. The *reliability* of the MSD scale, to assess the internal consistency of the scale as a whole - i.e., how closely related the set of MSD items are as a group. This analysis is based on the Cronbach’s Alpha statistic as well as on the Beta and Lambda coefficients; it is conducted as part of a Classical Test Theory (CTT) framework. This reliability analysis of the MSD scale as a whole is complemented with additional tests on the reliability of each individual item in the scale using Item Response Theory (IRT). Finally, a Hierarchical Omega Analysis is also presented.
4. The *additivity* of items, to test that the MSD indicator’s components add up – i.e. that someone with a MSD indicator score of “2” is suffering from more severe MSD than someone with a score of “1”. Additivity was measured for the MSD items that successfully passed the suitability, validity and reliability tests.

The MSD items that successfully passed these four steps can thus be considered to be robust candidates for being aggregated into a child-specific MSD indicator.

## Data on Children Deprivation in EU-SILC

The final list of items collected in 2009 that successfully passed the four tests in Guio et al. (2012) consists of 18 items, 13 child-specific items and 5 “household” items:

1. Child: Some new (not second-hand) clothes
2. Child: Two pairs of properly fitting shoes
3. Child: Fresh fruits and vegetables daily
4. Child: Meat, chicken, fish or vegetarian equivalent daily
5. Child: Books at home suitable for the children’s age
6. Child: Outdoor leisure equipment
7. Child: Indoor games
8. Child: Suitable place to do homework
9. Child: Regular leisure activities
10. Child: Celebrations on special occasions
11. Child: Invitation of friends to play and eat from time to time
12. Child: Participation in school trips and school events that cost money
13. Child: Holiday
14. Household: Arrears
15. Household: Home adequately warm
16. Household: Access to a car for private use
17. Household: Replace worn-out furniture
18. Adults in the household: Access to internet

Children MSD items are collected at the household level; they are not collected from the children themselves but from the adult answering the “household questionnaire” (i.e. the “household respondent”). According to the survey protocol, if, in a given household, at least one child does not have an item, it is then assumed that all the children belonging to that household lack that item. This assumption has been made for pragmatic reasons. Ideally, it would be preferable to know the deprivation levels of each child in a household separately as it would then be possible to study differences in child deprivation within each household as well as between households (e.g. if girls suffer more deprivation than boys, or teenagers more than younger children living in the same household). It would also be useful to know the views of children themselves about their living standards and confront these views with those of adults.

For most children’s items, the information was gathered for children aged between 1 and 15 (i.e. children’s items were collected in households with at least one child in this age bracket). Therefore, our suggested child-specific MSD indicator covers only children aged between 1 and 15.^5^ For consistency reasons, we had to exclude all children aged less than one from our calculations, even though information was available for some of them (where they have brothers/sisters aged between 1 and 15).

Two children’s items were collected only in households with at least one child attending school (school trips and suitable place to do homework) and are therefore less relevant for younger children. We have considered that children living in households where no child is attending school, by definition, do not lack these two items.

In the above list of items, contrary to some other analyses (see Gábos et al. 2011; de Neubourg et al. 2012; Watson et al. 2012; Whelan 2012), we have deliberately opted to complement the children’s items with some of the robust (i.e. suitable, valid, reliable and additive) MSD items collected at household level which do not refer explicitly to the situation of children but are known to affect children’s living standards. In line with scientific evidence, our choice is motivated by the fact that we strongly believe that, in order to adequately measure children’s MSD, it is necessary to look not only at those items that solely affect children but also at those that affect the households in which they live and that are likely to impact on their (current and/or future) living conditions. The whole set of items affecting children’s living conditions should then be included in a child MSD indicator, regardless of the statistical unit each individual item refers to (which, in many cases, primarily reflects a choice made on the basis of data collection rather than actual conceptual considerations). For example, a cold and/or damp home affects both children and adults – so it does not seem logical to only include this item as a measure of adult MSD and exclude it as a measure of child MSD.

As highlighted by Atkinson et al. (2002), the construction of indicators needs to follow a principle-based approach (see also Atkinson Commission on Global Poverty 2016); close links are required between the design of social indicators and the questions they are intended to answer. If the aim of the child MSD indicator is to measure intra-household transfers or within-household differences in living standards, then all household-level items would need to be removed from the MSD indicator. By contrast, if, as we want to do here, the objective is to measure and compare the living standards of children in different households, then the relevant household-level MSD items that have a direct effect on children’s living conditions need to be included in the child MSD indicator if they successfully pass our various robustness tests. This is particularly true where there is scientific evidence that these deprivations have worse or different effects on children than on adults (Marsh et al. 2000).

The inclusion of household items in a child indicator has to be interpreted from a *holistic and life-cycle* point of view. We consider items which directly and also indirectly impact on children’s living standards (e.g. direct items such as inadequate warmth in home, lack of durables etc.). Qualitative studies have also shown that children in households suffering from financial strain often do not ask their parents for the things they need which cost money in order to try to protect their parents from stress and feelings of guilt (Ridge 2002 and 2011; Observatoire de l’Enfance, de la Jeunesse et de l’Aide à la jeunesse, and Sonecom 2010). Thus we also include indicators of financial strain.

The 18 items selected by Guio et al. (2012) were collected for a second time in the 2014 wave of EU-SILC. The purpose of the next sections is to replicate and expand these analyses (Sections 4–7) to check whether all the 18 items also successfully pass the robustness tests in the 2014 dataset.

## Suitability

The analytical framework outlined in Section 2 requires the identification of items necessary to have a decent life in the society where people live. In the Townsend definition provided above, people are deprived if they lack the items “which are customary, or at least widely encouraged or approved, in the societies to which they belong”. Mack and Lansley (1985) proposed an innovative consensual approach to identify the “widely approved” necessities in Britain, by taking into account the judgment of individuals as to what constitutes an acceptable standard of living. They define necessities as possessions and activities that at least 50% of respondents regard as a necessity of life which everyone should be able to afford and no-one should have to do without. This approach has since been used in high-income countries (e.g. Van Den Bosch 2001; Halleröd 1995 and 2006; Saunders et al. 2007; Gazareth and Suter 2010; Abe and Pantazis 2013; Main and Bradshaw 2014) as well as middle-income and low-income countries (e.g. Kaijage and Tibaijuka 1996; Davies and Smith 1998; Ahmed 2007; Wright 2008; Mtapuri 2011; Nandy and Pomati 2015).

At the EU level, an EU wide Eurobarometer survey on the perception of poverty and social exclusion was carried out in 2007 (see TNS 2007 for a description of the survey). This Eurobarometer was the first EU dataset that allowed a comparative analysis of the items that citizens in the different Member States consider to be necessary for people to have an “acceptable” standard of living in the country where they live. The results of these analyses (see, for example, Dickes et al. 2010) led to the selection of a set of items, including children items that were included in EU-SILC.

In the absence of an up-to-date consensus survey (e.g. Eurobarometer) following the 2008 economic and financial crisis and the subsequent austerity measures, we used the actual behaviour of people, using EU-SILC data, to infer the degree of “desirability” associated to each item.

In EU-SILC, most MSD items distinguish between a “simple” lack of an item (children do not possess/ have access to the item) and an “enforced” lack of an item (parents would like their child(ren) to possess/ have access to the item but cannot afford it). For all children items (except the item related to the suitable place to do homework), three answer categories are used:

- have the item;
- do not have the item because cannot afford it;
- do not have the item for any other reason.

As Perry (2002) suggests, we define the desirability of each item, at EU and country levels, as the proportion of people “wanting” it - which encompasses both people who have (access to) the item AND people who would like to have (access to) it but cannot afford it. It has to be kept in mind that this relies on the assumption that *having* the item means *needing* it. We can argue that, at a certain level of living standard, people may have items they do not necessarily need but that are commonly possessed in the society where they live.

It is important to stress that our suitability test relies on indirect information about children’s needs, as parents reply to the questions about their children. Some authors suggest that parents may only imperfectly report on their children’s situation (for example, Ben-Arieh 2005) and that the views of adults and children about necessities can differ (Main and Pople 2011).

**Fig. 1:**
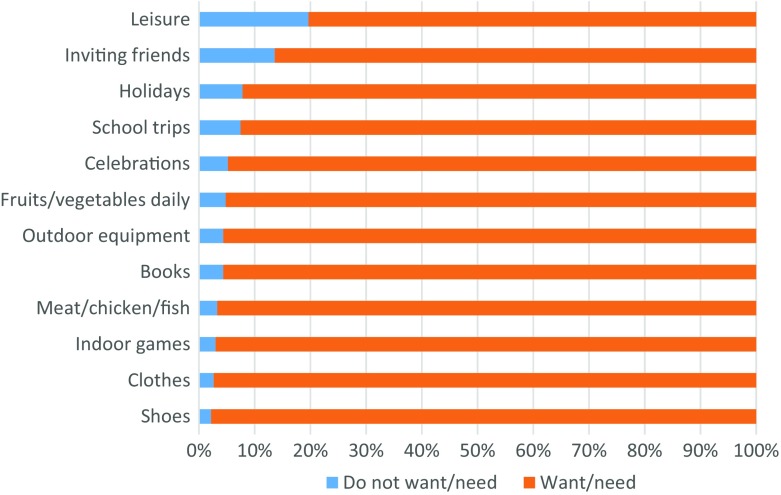
Children (aged between 1 and 15) (not) “wanting” the item, EU-28, 2014, (%). *Source*: EU-SILC 2014 cross-sectional data, authors’ computation. *Note*: People who “want” the item are people who have the item plus people who would like it but cannot afford it. By contrast, people who “do not want” the item are those who do not have it but for other reasons than lack of money

In our analysis, the proportion of children living with parents “wanting” the items is assumed to provide a measure of children’s ordinary living patterns, customs or activities which is a key criterion in Townsend’s sociological definition of poverty. For children’s MSD items, the proportion of children living with parents wanting each item is high, indicating a high degree of social consensus about these items (see Fig. 1 for EU aggregated results). This is true not only for basic items (food, clothes and shoes) but also for other items such as games, celebration, books or outdoor equipment. The items with the largest share of “not wanting/not having for other reasons” are the regular leisure activity (20%) and the possibility to invite friends round to play and eat (14%). All 12 children items that could be tested are highly suitable at EU aggregated level; this is also the case at the national level (results available on request). The 13th item, suitable place to do homework, could not be tested because the three answer categories required for the test are not collected in EU-SILC.

### Simple Lack or Enforced Lack?

In our analysis, only children suffering from “enforced lack”, i.e. lack due to lack of resources, are considered deprived. Those lacking the item “for other reasons” are treated, together with those who have the item, as not deprived. The “other reasons” modality potentially encompasses a large range of possible situations. If people who reply that their children do not have the item “for other reasons” do so for reasons positively correlated with their MSD level, children MSD rate will be underestimated and the analysis of child MSD may in turn provide erroneous information about the risk factors associated with MSD. Some authors therefore consider that any (simple) lack should be seen as MSD, whatever the reasons why the children lack the item. This makes sense in the specific case of children, on the grounds that children need all the items in the list (whatever the reasons for this need) and that they need to be protected as they do not always have a say in the decisions that concern them.

This choice may however be debatable for items that some children may really not want/ need (e.g. leisure, inviting friends, etc.). Considering “simple lack” instead of “enforced lack” then raises problems of comparability in relation to different preferences, which can reflect differences in culture, age and tastes. There are therefore strong theoretical reasons for preferring enforced lack to simple lack (Piachaud 1981; Mack and Lansley 1985).

Furthermore, consensus surveys tend to show that people’s perceptions of necessities do not support the hypothesis of adaptive preferences: those in greater financial difficulties are more likely to see items and activities as necessities (see for example Main and Bradshaw 2015, for a thorough analysis of the UK consensus survey on child deprivation).

Another interesting question is to know whether enforced or simple lacks are the best predictors of later-life-outcomes (educational attainment, health and income). The reply to this question depends on the children items which we focus on. For example, we could argue that simple lack (of adequate food or care) during childhood matters more than enforced lack to predict future health outcomes. Based on an analysis of the longitudinal data from the 1970 British Cohort Study, Diris and Vandenroucke (2016) argue that simple lack and enforced lack of durables (such as fridge, washing machine, dryer, TV, car, phone, video recorder etc.) are associated with later outcomes in a very similar way. Due to a range of limitations with these data these results should, however, not be generalised.

To better understand why people reply that they do not have an item for “other reasons”, we have estimated a multinomial logit model which predicts the probability of the three possible responses (i.e. have, do not have because cannot afford (enforced lack) and do not have because of other reasons) with a range of independent variables. Our results (available on request) show that there are non-negligible differences in terms of children’s age and country of residence in the probability of replying “other reasons” rather than “cannot afford”. Therefore, using the concept of enforced lack for the children items may help control for individual preferences due to different cultures, age and tastes.

In view of its importance, we come back to this issue of simple versus enforced lack in the next two sections. After testing the suitability (in the present section) of both concepts, we will test their validity (Section 5) and reliability (Section 6) so as to be in a position to make a final, evidence-based choice.

## Validity

Each item included in a MSD indicator needs to be a valid measure of MSD. An individual MSD item can be considered valid if it shows statistically significant associations with a set of variables known to be correlated with the latent construct of deprivation. We tested this by running binary logistic regressions for each of the 18 MSD items (dependent variable) against independent variables known to be correlated with MSD. Three indicators of validity were used:

- Income: there is a long tradition of using this association to validate MSD indicators. Both Peter Townsend (1979) and Mack and Lansley (1985) used the size of the correlation between income and deprivation to select their items. It is, however, well known that the overlap is far from perfect for a variety of reasons (Gordon et al. 2000; Berthoud et al. 2004; Halleröd 2006; Fusco et al. 2010).
- Subjective poverty (“great difficulties” or “difficulties” with making ends meet), which is often used as a measure of financial stress, is closely related to MSD (Fahmy and Gordon 2005; Nolan and Whelan 2007). It would be expected from Townsend’s theory of relative deprivation and Mack and Lansley’s (1985) concept of “consensual poverty” that someone “deprived” would be more likely to consider themselves to be subjectively poor (Bradshaw and Finch 2003). In our analysis, this would mean that we expect deprived children to be more likely to live in households where adults consider themselves to be poor.
- Household deprivation, measured with the EU “standard” material deprivation indicator that was used at the EU level up until March 2017 to measure deprivation for the whole population.^6^ We would expect that most MSD children live in a deprived household.

We consider there is a validity problem in a specific country when the country-level logistic regression odds ratios for at least two of the three variables (household deprivation, income and capacity to make ends meet) do not statistically significantly differ from one (no relation). Table 1 presents only the items with validity problems; all other items successfully passed the tests in all countries. The only country exhibiting a validity problem according to the criterion defined above is the Netherlands, where we find issues with availability of two items: suitable place to do homework and daily consumption of fruits/vegetables. This is partially driven by a small sample size problem.

**Table 1:** Items with validity problems in at least one country, Child population, 2014

	Household deprivation	Capacity to make ends meet	Log (income)	Not valid
Fruits & vegetables	Netherlands	Netherlands, Sweden	Finland	Netherlands
Meat			Finland	
Indoor games			Finland	
Outdoor equipment			Finland, Netherlands	
Friends		Finland		
Homework	Netherlands	Netherlands, Ireland	Netherlands	Netherlands
School trips			Netherlands	

*Source*: EU-SILC 2014 cross-sectional data, authors’ computation

We replicated these tests using the “simple lack” instead of the “enforced lack” concept. Our results show that the deprivation defined according to the enforced lack concept is more closely associated with the three variables used in the validity test, i.e. in most countries and for most items the odds are higher when using the enforced lack concept. So, using enforced lack rather than simple lack increases the validity of the index. Measures based on the enforced lack concept discriminate better between the worse-off and better-off children than those based on simple lack (for similar conclusions see Gordon 2006; Hick 2013).

## Reliability

Reliability was tested using Classical Test Theory (Section 6.1), Item Response Theory (IRT) models (Section 6.2) and Hierarchical Omega Analysis (Section 6.3).

### Classical Test Theory

#### Cronbach’s Alpha

The most widely used measure of reliability is the Cronbach’s Alpha statistic which measures the internal consistency of a scale, i.e. how closely related a set of items are as a group. A “high” value of Alpha is often used as evidence that the set of items measure an underlying (or “latent”) construct. An Alpha of 0.70 or higher is considered “satisfactory” in most social science research situations (Nunally 1978). We identified which items, if omitted (one by one), would increase the reliability of the deprivation index (i.e. increase Cronbach’s Alpha). This analysis was performed at both country and EU levels.

**Fig. 2:**
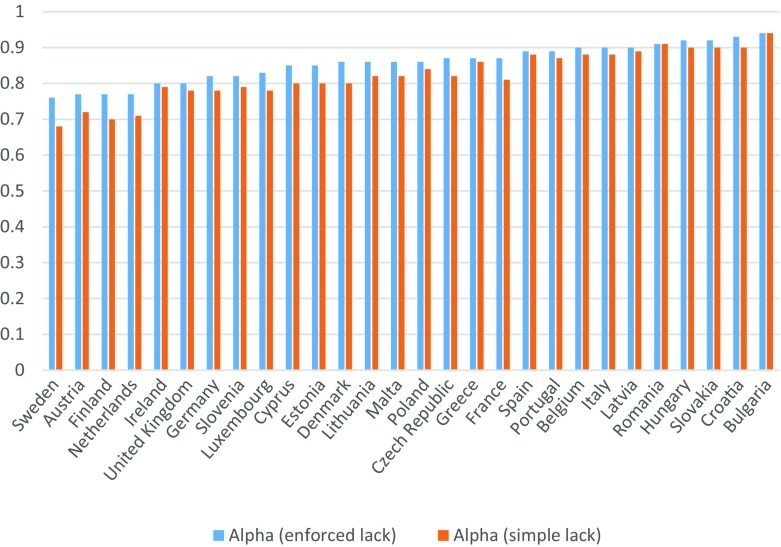
Cronbach’s Alpha, children items, National level, 2014. *Source*: EU-SILC 2014 cross-sectional data, authors’ computation

Examination of the detailed Alpha statistics for each item shows that the 18-item index would increase slightly in reliability in a few countries if certain items were removed. However, the gain in the overall reliability of the MSD index in each of these countries would be very small if these less reliable (but valid) items were dropped (see shaded cells in Table 2). The Cronbach’s Alpha using the “enforced lack” concept is greater than 0.70 in all EU countries and greater than 0.90 in seven countries (Fig. 2).

**Table 2:**
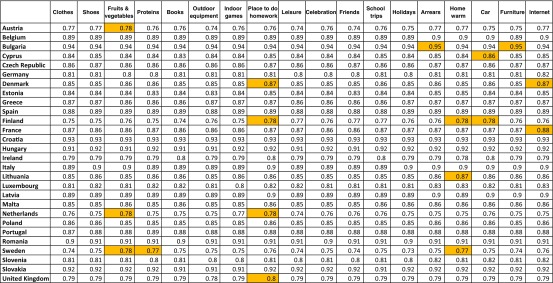
Cronbach’s Alpha, if the item is omitted, Child population, National level, 2014

*Source*: EU-SILC 2014 cross-sectional data, authors’ computation

The use of the “simple lack” concept decreases the reliability of the child MSD index, particularly in countries like Sweden and Finland where Cronbach’s Alpha is lower than 0.70 when the simple lack concept is used for children items. An indicator based on the enforced lack of the items is therefore more reliable than an indicator based on the simple absence of these items, as other authors have previously found (Halleröd 2006; Hick 2013). So, based on other research as well as our own results presented in this section and in Sections 4 and 5, the concept of enforced lack seems more appropriate for the measurement of child MSD: it leads to more valid and reliable measures and allows for differences in individual preferences due to different cultures, traditions and parental beliefs about the way to bring up children.

#### Beta and Lambda Coefficients

Although Cronbach’s Alpha is by far the most widely used measure of reliability, it has been criticised for failing to adequately estimate, under certain circumstances, the correct degree of reliability of a scale (Revelle and Zinbarg 2009). Classical Test Theory includes a number of reliability measures, each of which has its own strengths and weaknesses. In particular, Guttman (1945) derived six measures of the lower bounds of reliability – Lambda 1 to 6 (Alpha is equivalent to Guttman’s Lambda 3). Research has shown that, under certain conditions, Guttman’s Lambda 2 and Lambda 4 statistics produce more accurate estimates of the “true” reliability of an index than Cronbach’s Alpha (Jackson and Agunwamba 1977; Callender and Osburn 1979). We therefore analyse these two coefficients in this section.

**Fig. 3:**
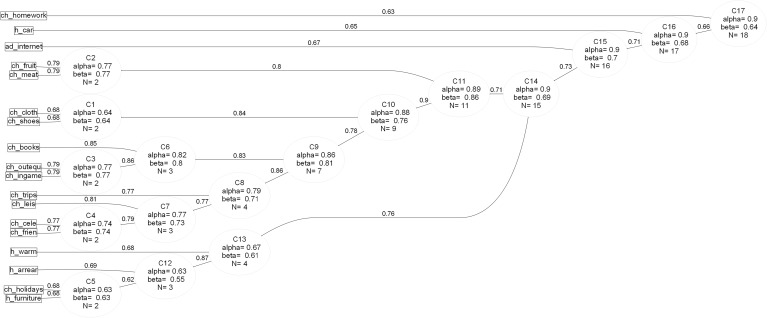
Item Cluster Analysis, Child population, EU level, 2014. *Source*: EU-SILC 2014 cross-sectional data, authors’ computation

We also analyse coefficient Beta, which can provide additional and complementary information about reliability. In particular, Alpha provides a good estimate of how well the MSD index will correlate with all other similar possible MSD indices with the same number of items, whereas Beta provides information about the homogeneity of the MSD index (e.g. if there are some items in the index which may be unreliable, causing the index to be “lumpy” (Revelle 1979)). Hierarchical cluster analysis permits the simultaneous analysis of the reliability and dimensional structure of an index using these two important reliability statistics (Beta and Alpha). It is a helpful technique to examine the way items are related according to their angular proximity (i.e. correlation) and how reliable the sub-groupings are. Both Beta and Omega (see Section 6.3 below) can be considered to be estimates of the percentage of the index that measures a single latent construct – for instance, deprivation. Figure 3 shows the results of the Item Cluster Analysis for the whole set of countries. According to this analysis, the 18 items can be grouped into 17 reliable clusters. The last item grouped with others (suitable place to do homework, cluster 17) decreases the Beta coefficient, indicating that this is the least reliable item. However, at EU level all MSD items have a Beta above 0.5 indicating that they are all reliable measures of child MSD.

Focusing on the results at the national level, Table 3 shows the Beta, Lambda 2 and Lambda 4 statistics for the items included in the child-specific MSD scale in each EU Member State. In all Member States, the Guttman’s Lambda 2 and 4 coefficients are greater than the threshold value of 0.7 and for most countries above 0.8 – indicating a very reliable MSD measure. Looking at the Beta value enables judgments about the internal structure of the index and about the possible existence of sub-groups. Results show that half the countries have a low value (Beta <0.5) which may indicate some homogeneity problems in these countries, e.g. the index may include some items that are not strongly correlated with the rest.

**Table 3:** Beta, Lambda 2 and Lambda 4 coefficients, Child population, National level, 2014

	Beta	Lambda 2	Lambda 4
EU-28	0.61	0.90	0.93
Austria	0.41	0.76	0.83
Belgium	0.58	0.91	0.94
Bulgaria	0.61	0.94	0.96
Cyprus	0.3	0.86	0.9
Czech Republic	0.41	0.86	0.91
Germany	0.55	0.82	0.87
Denmark	0.39	0.85	0.91
Estonia	0.48	0.82	0.89
Greece	0.62	0.87	0.91
Spain	0.57	0.89	0.92
Finland	0.5	0.74	0.85
France	0.47	0.86	0.9
Croatia	0.57	0.91	0.94
Hungary	0.75	0.92	0.95
Ireland	0.51	0.82	0.88
Italy	0.37	0.9	0.93
Lithuania	0.42	0.86	0.91
Luxembourg	0.42	0.85	0.9
Latvia	0.63	0.89	0.93
Malta	0.6	0.87	0.91
Netherlands	0.12	0.78	0.85
Poland	0.58	0.87	0.9
Portugal	0.68	0.87	0.91
Romania	0.06	0.91	0.94
Sweden	0.23	0.79	0.88
Slovenia	0.45	0.83	0.88
Slovakia	0.58	0.92	0.94
United Kingdom	0.4	0.8	0.85

*Source*: EU-SILC 2014 cross-sectional data, authors’ computation

Further tests (available on request) show that, in most cases, the drop in reliability is due to just one item. If this item is removed, then the Beta value becomes higher than 0.5. In most countries, the item that shows reliability problems is the enforced lack of a suitable place to do homework (Czech Republic, Denmark, Estonia, Netherlands, Lithuania, Romania, the UK). In Cyprus, Denmark, Italy and Slovenia, the enforced lack of a car is problematic, which is a well-known result and is due to the very high car possession rate in these countries. Similarly, the capacity to keep the home adequately warm in Finland, Sweden and Lithuania shows reliability problems, a result due to the need of all households (including the most deprived ones) to heat their homes in very cold climatic conditions.

### Item Response Theory

Item Response Theory (IRT) consists of a set of statistical models which describe the relationship between a person’s response to questionnaire items and an unobserved “latent trait” such as knowledge of science, degree of happiness or level of MSD. It is often used for the selection of questions in educational assessment and for psychological testing. It has also been used for developing measures of poverty (e.g. Cappellari and Jenkins 2007; Fusco and Dickes 2008; Martini and Vanin 2013; Szeles and Fusco 2013).

In our analysis, we applied a two-parameter IRT to test each of the 18 MSD items. The first parameter can be interpreted as the likely severity of MSD suffered by a child who lacks this item because he/she cannot afford it (“enforced lack”). The severity scores are measured in units of standard deviation from the average child. We set the severity criterion at 3 standard deviations from the mean, i.e. we flag all items with a severity greater/ lower than 3 standard deviations (severity levels between 3 and 3.5 are considered “borderline”). At EU level, all items pass this test. At the national level, there are a few items that have severity problems. The item related to the lack of a suitable place to do homework is associated with (extremely) high levels of MSD in 15 EU countries, which means that it is likely to affect only a very small number of children (those who have a level of MSD higher than 3 standard deviations). This makes it unsuitable for the reliable measurement of MSD in surveys with relatively small sample sizes. Keeping the home adequately warm is associated with severe deprivation in Finland, Estonia and Sweden (these are all Northern European countries where, as we argued above, keeping the home warm is a matter of survival) and also in Luxembourg. Internet access is associated with high deprivation levels in Sweden and the Netherlands. It is borderline in Denmark, Finland and Luxembourg (these are all countries where internet access is close to saturation).^7^ The lack of school trips for affordability reasons is associated with very severe deprivation in Germany and the Netherlands (and is borderline in Austria, Denmark, Finland and Slovenia); this result has to be compared with information on the school costs in the different countries. Finally, the lack of fruits and vegetables is very severe in three countries (Austria, Netherlands and Sweden) and borderline in Denmark.

The second parameter is the discrimination of the item. It indicates how well each item discriminates between the deprived and non-deprived children, and it can be transformed into a correlation coefficient (ranging from −1 to +1) between each item and MSD. The discrimination criterion we use here is to highlight all items whose correlation with MSD is lower than 0.4. At EU level, all items pass this test. At the national level, keeping the home adequately warm in Lithuania and suitable place to do homework in Romania and the Netherlands have a correlation with the overall latent trait of deprivation below 0.4.

**Fig. 4:**
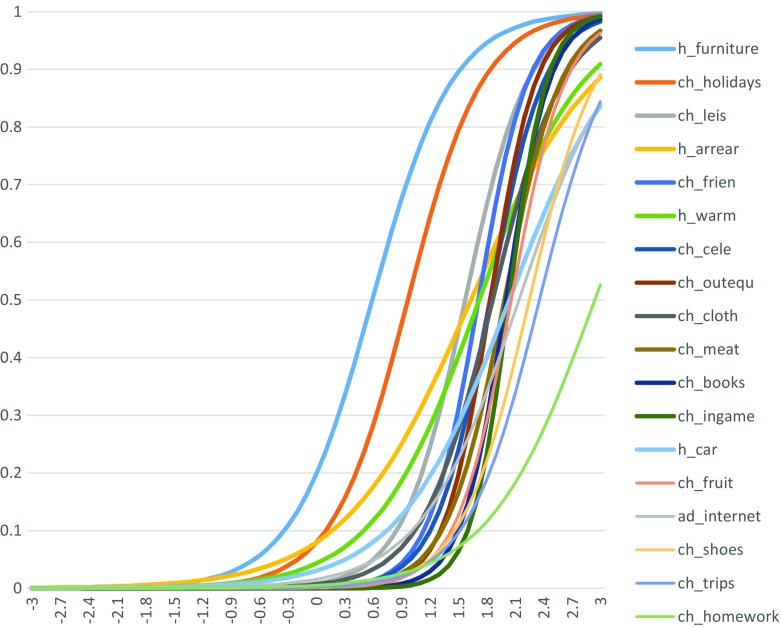
Item Characteristic Curves, Child population, EU level, 2014. *Source*: EU-SILC 2014 cross-sectional data, authors’ computation. *Note*: Legend sorted by item severity (from least severe MSD items to most severe MSD items)

Item Characteristic Curves illustrate both the discrimination and the severity of each item (see Fig. 4). The severity of each item is shown by the position of each asymptotic (i.e. “S” shaped) curve along the X-axis – the further to the right, the more severe the deprivation. The ability of each item to discriminate between the deprived and non-deprived people/households is shown by how vertical each curve is with respect to the y-axis. The more upright it is, the better the discriminating ability of the item and the higher its correlation with MSD.

Ideally, a “good” MSD scale would be illustrated by a series of fairly vertical “S” shaped curves spread out along the X-axis. The inflection point of each curve, that is, half the distance between the upper and lower asymptotes, where the slope is steepest, should lie between 0 and +3 on the X-axis. In other words, it should have a severity of between 0 and +3 standard deviations. As shown in Fig. 4, the lack of a suitable place to do homework stands out as the item which conforms less to the ideal pattern – which is consistent with our above results.

### Reliability: Hierarchical Omega Analysis

Despite its popularity and widespread use in scale development, the Cronbach’s Alpha coefficient has some potential drawbacks and limitations (see discussion in Section 6.1.2). The Omega statistic ω and the Omega Hierarchical statistic ωh (both range between 0 and 1) can produce a more accurate estimate of the reliability of the MSD scale, which corresponds to the greatest lower bound of reliability (Revelle and Zinbarg 2009). ω is a measure of overall reliability and ωh is a useful reliability statistic when a measure is multidimensional (Zinbarg et al. 2005). For example, it is arguable that an overall MSD index may contain two sub-dimensions such as material deprivation and social deprivation. ωh estimates the proportion of overall variation in the MSD items (whether material or social) accounted for by an overall deprivation dimension (a higher-order factor).

To calculate ω and ωh, two measurement models were estimated: one assuming unidimensionality and one drawing from the theoretical work of Townsend (1979, 1987):

- Model 1: Unidimensional model.
- Model 2: Townsend model. A simplified version of Townsend’s deprivation structural model where deprivation (the higher order-factor) comprises two dimensions: material and social deprivation.^8^

The main purpose of the Omega analysis is twofold. First, it compares the unidimensional model with the structural model drawn from relative deprivation theory, and it shows the extent of multidimensionality of the list of items. Secondly, it estimates both the Omega and the Hierarchical Omega statistics to assess the reliability and importance of the higher order factor (overall deprivation), as presented in Table 4.

**Table 4:** Child omega analysis, Child population, National level, 2014

	Unidimensional		Townsend		
	Omega	BIC	Omega	Hierarchical Omega	BIC
EU-28	0.97	2,968,276	0.96	0.65	2,995,327
Austria	0.97	36,513	0.95	0.64	36,672
Belgium	0.98	54,206	0.97	0.66	55,170
Bulgaria	0.97	121,397	0.96	0.66	123,148
Cyprus	0.97	74,146	0.95	0.64	74,961
Czech Republic	0.97	73,612	0.96	0.65	74,221
Denmark	0.97	37,939	0.97	0.66	38,207
Germany	0.97	80,486	0.96	0.65	80,840
Estonia	0.97	63,526	0.96	0.65	63,475
Greece	0.96	159,340	0.95	0.65	160,420
Spain	0.98	165,461	0.97	0.66	167,869
Finland	0.98	56,505	0.97	0.66	56,843
France	0.97	98,798	0.96	0.65	99,671
Croatia	0.98	74,733	0.97	0.66	75,166
Hungary	0.97	165,523	0.97	0.65	167,176
Ireland	0.96	75,822	0.95	0.64	76,181
Italy	0.98	243,163	0.97	0.65	244,952
Lithuania	0.97	69,019	0.96	0.65	69,735
Luxembourg	0.98	23,481	0.97	0.66	23,892
Latvia	0.97	98,751	0.96	0.65	99,357
Malta	0.97	59,405	0.96	0.65	59,819
Netherlands	0.96	50,871	0.96	0.65	51,623
Poland	0.97	185,147	0.96	0.65	186,599
Portugal	0.97	105,219	0.96	0.65	106,230
Romania	0.96	196,129	0.94	0.66	198,337
Sweden	0.98	18,938	0.95	0.64	19,361
Slovenia	0.97	108,931	0.96	0.64	109,721
Slovakia	0.97	84,723	0.96	0.65	85,340
United Kingdom	0.96	107,076	0.95	0.64	107,615

*Source*: EU-SILC 2014 cross-sectional data, authors’ computation

*Note*: BIC stands for Bayesian Information Criteria

This table contrasts the global statistic of fit (adjusted Bayesian Information Criteria (BIC)) of the two structural models. The unidimensional model has a slightly better fit compared with the Townsend model. This result questions the current methodology of UNICEF’s rights-based approach to multidimensional child poverty measurement in the EU, which consists of aggregating children MSD items first by sub-dimensions (through a normative child rights lens) and then across sub-dimensions (see for example de Neubourg et al. 2012; Chzhen et al. 2017). Our results indicate that this might not be the best option and that the reliability of the UNICEF model would need to be tested in view of the fit of the unidimensional model.

At the national level, Table 4 shows that the Omega coefficient (unidimensional model) is higher than 0.96 in all EU countries, which is an excellent result. The results from the Townsend model also suggest that the overall (higher order) deprivation factor accounts for more than half (around 65%) of all the variance in the deprivation items.

## Additivity

Additivity tests aim to ensure that the MSD indicator’s components add up, i.e. to check that, say, someone with a MSD indicator score of “2” is in reality suffering from more severe MSD than someone with a score of “1” or a score of “0”. This was checked using an ANOVA model (second order interactions of MSD items by level of equivalised disposable household income). Negative incomes were adjusted according to the methodology proposed by Verma (2007). These additivity models test whether children who suffer from two deprivations (e.g. those who cannot afford both clothes *and* shoes) live in households with (on average) significantly lower net equivalised incomes than those who “only” suffer from one deprivation (clothes *or* shoes deprivation only) or no deprivations. Similarly, those children suffering from one deprivation would be expected to have lower equivalised household incomes than those with no deprivations. This should hold for all possible combinations of deprivation items.

For the 28 EU Member States, only 17 statistically significant (but still fairly minor) interaction problems were detected. Some of them were probably due to there being too few cases of joint deprivation to accurately calculate 95% confidence intervals of the mean household income and/or the presence of an “outlier” in one of the categories. For example, the item related to the enforced lack of school trips shows additivity problems in Germany but only 0.7% of the children suffer from enforced lack of school trips in this country. Similarly, the two interaction problems in Denmark concern items lacked by around 0.5% of the children.

## Final List of Children Items

Table 5 summarises the results of the various tests performed on the data. The item related to a suitable place to do homework does not pass our reliability tests and has therefore to be dropped from the list. Due to the impact of educational deprivation on adult outcomes, we want to highlight the importance of replacing this item in future data collection by alternative items which measure educational deprivation.

**Table 5:**
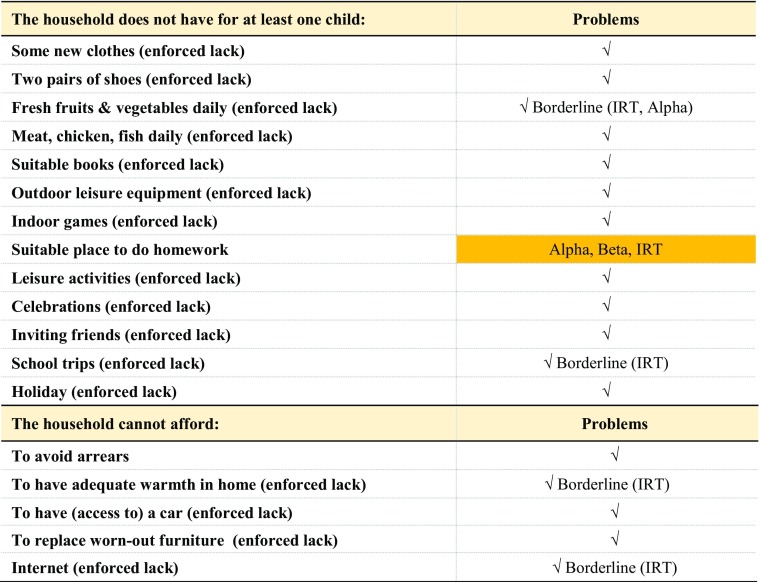
Outcomes of suitability, validity, reliability and additivity tests, Child population, 2014

*Note*: √ = successful on all criteria

*Source*: EU-SILC 2014 cross-sectional data, authors’ computation

The incidence of each individual MSD item retained in our proposed final child MSD list is presented in Table 6 and compared to the EU-28 average. This heat map highlights countries showing consistently high MSD levels across several items such as Bulgaria and Romania.

**Table 6:**
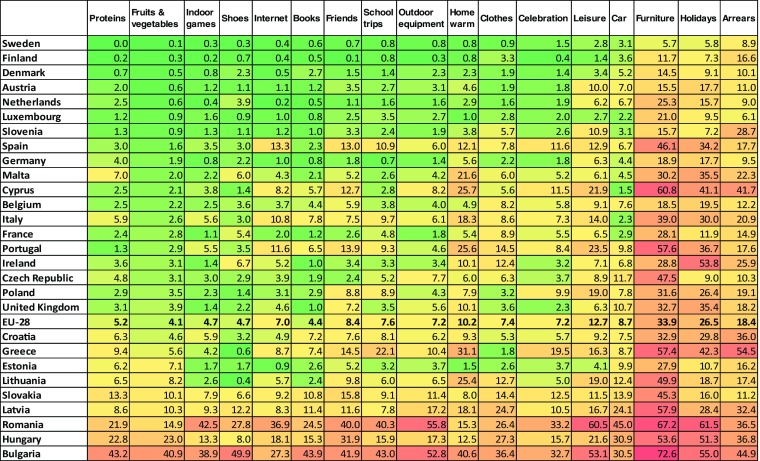
“Heat map” providing for each item the proportion of children lacking the item in the country, Child population, National results, 2014

*Source*: EU-SILC 2014 cross-sectional data, authors’ computation

**Fig. 5:**
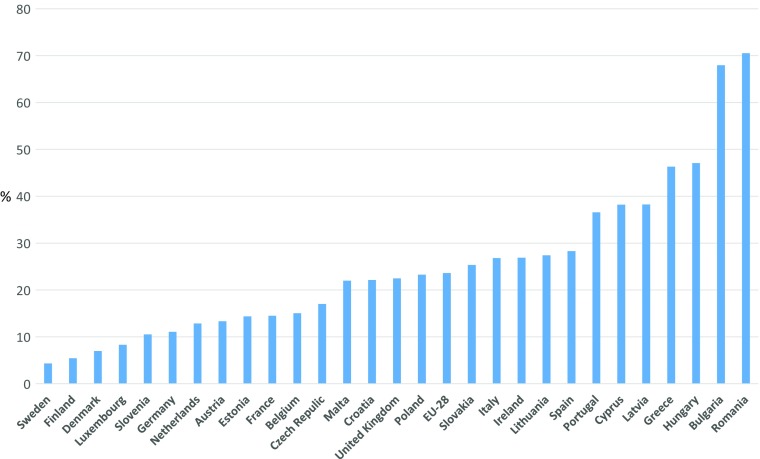
Children lacking at least three out of 17 items, National results, 2014, (%). *Source*: EU-SILC 2014 cross-sectional data, authors’ computation

We tested different thresholds for the child MSD index. For illustrative purposes, Fig. 5 provides the distribution of national MSD rates calculated on the basis of a threshold set at three out of 17 deprivations. National proportions of deprived children vary hugely across EU countries, from 5 to 10% in Sweden, Finland, Denmark, Luxembourg and Slovenia to around 70% in Bulgaria and Romania.

## Conclusions

This paper has proposed a careful and systematic analytical framework to identify an optimal set of robust items to be included in an EU child-specific MSD indicator for use by the European Commission and Member States in their regular social monitoring. As a result of this analysis, 17 items have been retained; each of them provides a suitable, valid, reliable and additive measure of MSD in almost all EU Member States. Some of the items are however borderline in a few countries. In particular, some items are associated with very severe deprivation in some countries (keeping the home warm in Nordic countries and lack of internet in countries where this is close to saturation). Yet, their exclusion from the list would have virtually no impact on the child-specific MSD rate in these countries as the proportion of children lacking them is (extremely) low. However, given that these items do discriminate between deprived and non-deprived children in the other countries, we would advise that the list of items is kept identical in all the Member States so as to obtain an easily comparable child MSD indicator for the whole EU.

The very high level of reliability of the final list needs to be highlighted. These results have been stable over time (between the 2009 and 2014 EU-SILC waves) except for the “suitable place to do homework” which had passed the tests with the 2009 data but had to be dropped from the final list after failing to pass the reliability tests applied to the 2014 data. All the 17 retained child MSD items were found to be suitable, valid, reliable and additive in both 2009 and 2014.

In order to avoid underestimating child MSD, we tested the validity and reliability of both the “simple” and “enforced” lack concepts. Our results indicate clearly that the enforced lack concept discriminates better between the worst-off and the better-off children and leads to more valid and reliable indices. It also allows for differences in parental preferences due to the age of the child, the national context (different cultures and traditions) and beliefs about the ways to bring up children.

Another important question we addressed in the paper is the extent of uni−/multi-dimensionality of the EU child MSD index. Many authors aggregate items across sub-dimensions first and then aggregate sub-dimensional results into one single measure. When doing so, they assume that the index is multidimensional. Our Omega analysis shows that this hypothesis may not be correct as the unidimensional structural model we tested slightly outperforms the simplified Townsend model, which assumes two sub-dimensions.

Our proposed indicator is based on the unweighted sum of the 17 MSD items for each child. It is self-evident that some MSD items are more important than others. However, the consistently high levels of reliability of this indicator suggest that no set of item weights (even if error-free) would, when applied to these deprivation items, lead to an index that represents child deprivation more accurately (Kline 2005).

In March 2017, the EU adopted a new indicator of “material and social deprivation”. This measure, which was developed by Guio et al. (2012, 2016) covers the entire population. In this paper, we have shown that it is eminently feasible to produce a suitable, valid, reliable and additive deprivation measure focused on the specific situation of children using EU-SILC data. We hope that the EU will adopt such a child MSD measure in the near future. It would be an important step in the direction of the EU Social Protection Committee’s commitment to including (at least) one indicator on “child well-being” in the EU portfolio of social protection and social inclusion indicators and to improve the EU toolbox needed for monitoring progress in the implementation of the EU Recommendation on “Investing in Children: breaking the cycle of disadvantage” endorsed by all EU countries in 2013.

## Footnotes

1 This study ran in over 50 low and middle income countries and adopted the multidimensional child deprivation measure proposed by Gordon et al. (2003). See: https://www.unicef.org/socialpolicy/index_45357.html.

2 The most recent EU objectives for social protection and social inclusion were agreed in 2011 (Council of the European Union 2011). A set of commonly agreed EU social indicators is used for monitoring progress towards these objectives. This set is continuously fine-tuned and complemented with new measures. The EU body in charge of developing these EU social indicators is the Indicators Sub-Group of the EU Social Protection Committee (http://ec.europa.eu/social/main.jsp?catId=830&langId=en). On the use of EU social indicators and the methodological EU framework under which these are developed, see also: Atkinson et al. (2002) and Marlier et al. (2007).

3 These recommendations were grouped into six categories: setting quantified objectives, assessing the impact of policies on child poverty and social exclusion, monitoring child poverty and well-being, common framework for analysing and monitoring child poverty and social exclusion, reinforcing statistical capacity, and improving governance and monitoring arrangements at all relevant policy levels.

4 After the 2005 Luxembourg EU Presidency, three EU Presidencies played an instrumental role in this context. These are the Presidencies held by Belgium (2010), Hungary (2011) and Cyprus (2012).

5 It is important to highlight that, as a result of the way data were collected in the 2009 and 2014 EU-SILC ad hoc modules on deprivation, “children” here do not refer to the same population as the one covered by the existing EU social indicators: here, children are individuals aged 1–15 as opposed to 0–17 (in EU-SILC, teenagers aged 16 and 17 are interviewed individually on the basis of the adult questionnaire).

6 The “standard” EU material deprivation indicator was defined as the proportion of people living in households confronted with at least three of the following nine lacks (see Guio 2009): 1) they cannot face unexpected expenses; 2) they cannot afford one week annual holiday away from home; 3) they cannot avoid arrears (in mortgage or rent, utility bills and/or hire purchase instalments); 4) they cannot afford a meal with meat, chicken, fish or vegetarian equivalent every second day; 5) they cannot keep their homes adequately warm; 6) they cannot afford to have access to a car/van for personal use; 7) they cannot afford a washing machine; 8) they cannot afford a colour TV; and 9) they cannot afford a telephone. This indicator was referred to as “standard” material deprivation as opposed to the “severe” material deprivation (threshold of four deprivations out of nine) included in the Europe 2020 “at-risk-of-poverty-or-social-exclusion” target, which is based on an aggregate indicator consisting of the union of this severe deprivation measure, an income poverty measure and a measure of (quasi-)joblessness (see Frazer et al. 2014 for a discussion of this target). In March 2017, the European Commission and all EU countries decided to replace the “standard” deprivation indicator with a new indicator based on the work by Guio et al. (2012 and 2016). The new indicator consists of 13 items: seven household deprivation items (items 1–6 of the previous “standard” deprivation indicator plus incapacity to replace worn-out furniture) and six personal deprivation items (inability for the person to: replace worn-out clothes with some new ones, have two pairs of properly fitting shoes, spend a small amount of money each week on him/herself, have regular leisure activities, get together with friends/family for a drink/meal at least once a month, and have an internet connection). Referred to as “Material and social deprivation rate”, this indicator is now included in the portfolio of EU social indicators used by the Commission and Member States to monitor EU progress towards the EU social protection and social inclusion objectives. The new indicator covers the entire population.

7 The item is also borderline in the UK, which is likely to be due to problems of data collection.

8 In the 1968/69 Poverty in the United Kingdom study, Townsend divided “deprivation” into these two major categories (Material and Social) and further sub-divided them into seven and four sub-groups, respectively (see Townsend 1979, pp.1173–1176). Townsend refined his deprivation model during his development work on the 1987 Booth Centenary Survey of Poverty and Labour in London (Townsend 1987; Townsend and Gordon 1989).
